# Cross-Modal Contrastive Hashing Retrieval for Infrared Video and EEG

**DOI:** 10.3390/s22228804

**Published:** 2022-11-14

**Authors:** Jianan Han, Shaoxing Zhang, Aidong Men, Qingchao Chen

**Affiliations:** 1 School of Artificial Intelligence, Beijing University of Posts and Telecommunications, Beijing 100876, China; 2 Peking University Third Hospital, Beijing 100191, China; 3 National Institute of Health Data Science, Peking University, Beijing 100191, China

**Keywords:** infrared video, EEG, sleep quality, sleep apnea syndrome, cross-modal retrieval, telemedicine, monitoring of follow-up clinical interventions, management of chronic diseases

## Abstract

It is essential to estimate the sleep quality and diagnose the clinical stages *in time* and *at home*, because they are closely related to and important causes of chronic diseases and daily life dysfunctions. However, the existing “gold-standard” sensing machine for diagnosis (Polysomnography (PSG) with Electroencephalogram (EEG) measurements) is almost infeasible to deploy at home in a “ubiquitous” manner. In addition, it is costly to train clinicians for the diagnosis of sleep conditions. In this paper, we proposed a novel technical and systematic attempt to tackle the previous barriers: *first*, we proposed to monitor and sense the sleep conditions using the infrared (IR) camera videos synchronized with the EEG signal; *second*, we proposed a novel cross-modal retrieval system termed as Cross-modal Contrastive Hashing Retrieval (CCHR) to build the relationship between EEG and IR videos, retrieving the most relevant EEG signal given an infrared video. Specifically, the CCHR is novel in the following two perspectives. *Firstly*, to eliminate the large cross-modal semantic gap between EEG and IR data, we designed a novel joint cross-modal representation learning strategy using a memory-enhanced hard-negative mining design under the framework of contrastive learning. *Secondly*, as the sleep monitoring data are large-scale (8 h long for each subject), a novel contrastive hashing module is proposed to transform the joint cross-modal features to the discriminative binary hash codes, enabling the efficient storage and inference. Extensive experiments on our collected cross-modal sleep condition dataset validated that the proposed CCHR achieves superior performances compared with existing cross-modal hashing methods.

## 1. Introduction

According to recent scientific findings [1,2], millions of people with chronic and psychiatric diseases have sleep-related problems, that are highly correlated with daily life dysfunction and even traffic accidents. As we human beings approximately spent 8 h in sleep at home per day, it brings significant benefits if a ubiquitous sleep monitoring technique is available for diagnosis both at home and in time. *However,* clinicians currently identify sleep conditions using the complex and expensive polysomnography (PSG) machine which is challenging to deploy at home.

To solve the previous barrier of ubiquitous sleep monitoring, we propose to use the easy-to-deploy infrared (IR) camera sensor (IR videos) to monitor sleep quality with the help of the synchronized clinical PSG machines (the Electroencephalogram (EEG) signal). In this paper, we adopt the methodology to retrieve the EEG signal based on the query of the IR video, by modelling the relationship between descriptors of the IR video and the EEG signal. The background intuition is to sense the sleep quality at home using the easy-to-deploy IR sensor *but* retrieve the relevant EEG signal for fine-grained diagnosis.

Accurate sleep classification results are essential for analyzing sleep stages and diagnosing Obstructive Sleep Apnoea (OSA). PSG is recognized as the “gold-standard” for sleep stage classification by the American Academy of Sleep Medicine (AASM) and Sleep Clinical Medicine. Additionally, the PSG label is generated based on a combination of multi-leads (relying primarily on EEG leads) and supplemented by human fine-tuning by at least three physicians. The physicians’ fine-tuning process is to view the infrared video information corresponding to the questionable classification results. In other words, the infrared video is crucial for the final labeling of the sleep stage classification labels. The results of previous studies [3,4,5] show a relatively high accuracy of the results of sleep stage classification using machine learning methods by single-channel EEG. Additional findings suggest [6,7,8,9,10,11] that video information also plays an essential role in sleep stage classification and quality analysis. Specifically: ref. [6] shows that by counting the sleep movements of 11 healthy adults over about 30 days, the frequency of physical activity could be summarized as *W* > N1 > REM > N2 > N3. Body movements during sleep and brief awakenings are directly related to the perceived quality and depth of sleep [10]. Some sleep disorders, such as periodic limb movement disorder or rapid eye movement (REM) sleep behavior disorder, are characterized by major or minor movements [11]. When a doctor diagnoses a patient, he often compares “certain features” of the patient he has previously diagnosed in his mind, a process similar to retrieval. Since IR video is easier and more painless to obtain than EEG, we are considering designing a cross-modal retrieval algorithm to perform mutual retrieval between IR video and EEG to help doctors use IR video for initial diagnosis.

To our best knowledge, we have not found works that investigated the cross-modal retrieval task between IR video and the EEG signals. As a pioneering work, we posited the following two challenges:

**1**: The semantic gap between IR video and EEG signal is large compared to other cross-modal retrieval tasks. It brings challenges to capture consistent cross-modal semantics in the retrieval task.
**2**: Sleep data are large-scale (especially IR video), and require large storage for the gallery sets and superior computing resources in the inference.

If the successful diagnosis or cure of similar cases in the past can be used as a reference for treating new cases, the success rate and efficiency of sleep-related treatment will be greatly improved; this process coincides with the idea of retrieval. So it is imperative to complete the cross-modal retrieval between infrared video modality and EEG modality.

As far as we know, there are few methods to study the cross-modal retrieval of sleep IR video and EEG signals, mainly because of the following two challenges: (1) The semantic gap between IR video and EEG is large compared to other cross-modal retrieval tasks; capturing semantic consistency between modalities is more critical. (2) Due to the particularity of sleep data (especially infrared video), it requires a lot of storage space and computing resources.

In this paper, we propose a novel Cross-modal Contrastive Hashing Retrieval (CCHR) method to address the above two challenges. We highlighted our contributions as follows:

- To reduce the large cross-modal semantic gaps, we designed a contrastive learning method based on hard negative samples, that pulls closer the inter-modal similar representations and pushes the dissimilar ones.
- To solve the problem of excessive sleep data storage, we proposed a novel contrastive hashing module to compute a discriminative yet unique cross-modal binary hash codes.
- For evaluations, we collected a large-scale synchronized IR video and EEG data from the clinics. Results proved that our proposed CCHR significantly outperforms the current state-of-the-art cross-modal hashing retrieval methods.

## 2. Related Works

### 2.1. Feature Representation for Video-EEG Retrieval

#### 2.1.1. EEG

Traditional manual sleep staging methods rely on the observations of experienced physicians [12], supplemented by analytical adjustment methods. In this way, the most experienced physicians take several hours to annotate a patient’s data, making it difficult to ensure accuracy and annotation efficiency. Machine learning-based sleep staging methods are mainly based on support vector machines (SVM) [13] and random forests (RF) [14]. In contrast, with the popularity of deep learning methods in recent years, more and more EEG-based sleep staging methods [15,16,17,18,19,20,21,22,23] have become mainstream. Deepsleepnet [3] is a new model architecture that uses two CNNs with different filter sizes and a bi-directional LSTM in the first layer to extract time-invariant features from the original single-channel EEG. SleepEEGNet [24] uses CNNs to extract time-invariant features, frequency information and sequence-to-sequence features to capture the complex and long-term short-term contextual dependencies between sleep epochs and scores. MultitaskCNN [25] is a multi-task CNN framework for automatic sleep staging that introduces a joint classification and prediction formulation. It can jointly perform sleep stage classification of input epochs and predict the labels of their neighbors in the contextual output. Attnsleep [5] is a new attention-based deep learning architecture that uses a multi-resolution convolutional neural network (MRCNN) and an adaptive feature recalibration (AFR) feature extraction module to classify single-channel EEG signals during the sleep phase. GraphSleepNet [26] proposes a new deep graph neural network that adaptively learns the intrinsic connections between different electroencephalographic (EEG) channels and uses them for automatic sleep stage classification. Jia et al. [27] proposed a salient multimodal wave detection network SalientSleepNet for sleep staging. SalientSleepNet can efficiently detect and fuse salient waves in multimodal data and extract multi-scale transition rules between sleep stages.

#### 2.1.2. Video

For video feature representation, early works often extract hand-crafted visual features by computing dense trajectories [28], SIFT-3D [29], and HOG-3D [30]. SlowFast [31] characterizes the variation within each video compactly and provides fixed length of representation for a video with any number of frames. DHH [32] uses a covariance matrix to model face video and achieves good results in image-video retrieval. Hara et al. proposed 3D Resnet [33], which uses 3D convolution to model video information and is pre-trained on large-scale datasets containing approximately 100k videos. Du Tran et al. proposed R(2+1)D architecture [34], that separately computes the spatial and temporal features for model efficiency. We adopt the 3D Resnet-18 as our IR video encoder to obtain a powerful video representation in this work.

### 2.2. Cross-Modal Contrastive Learning

Cross-modal research has attracted a lot of attention recently [35,36,37,38,39,40,41,42], especially some video-related work [43,44], which has achieved good results. Additionally, with the great success of contrastive learning in the field of unsupervised representation learning, more and more researchers are applying contrastive learning methods to cross-modal studies [45,46,47,48]. Li et al. [45] proposed a unified model pre-training architecture, UNIMO, which can be efficiently adapted to uni-modal and multi-modal comprehension and generation tasks using contrastive learning. Kim et al. [46] proposed a new adaptive framework for multi-modal video domains that exploits features in four different feature spaces across modalities and domains with promising results. XMC-GAN [47] uses a simple single-stage GAN that employs several contrastive losses to accomplish text-to-image generation. CrossCLR [48] presents a contrastive loss for learning joint embeddings of two input modalities that respects the special need of cross-modal learning.

### 2.3. Contrastive Learning for Cross-Modal Retrieval

The contrastive learning framework is effective in retrieval tasks, that aims to learn an embedding space where similar samples are close to each other while dissimilar samples are far apart [49]. InfoNCE [50] inherits the basic idea of NCE, introduces negative examples from a new distribution, constructs a new multivariate classification problem, and proves that reducing this loss function is equivalent to increasing the lower bound of mutual information. Hu et al. used contrastive learning to design a simple and effective multimodal loss function, called multi-modal contrastive loss (Mc), that maximizes the use of mutual information between different modes, thus reducing noise interference and inter-modal difference. In the cross-modal retrieval method DUCH [51], the normalized temperature-scaled cross-entropy is proposed in [52] as a novel contrastive loss. In our work, due to the specificity of the context of our task (continuous and similar infrared sleep videos), to improve the sensitivity to contrastive loss, we only use the “hardest” (distance closest and label inconsistent) rather than the whole group when selecting negative samples for comparison.

### 2.4. Hashing Methods for Cross-Modal Retrieval

To meet the requirements of low storage cost and high inference speed, the hashing based retrieval has become an important research direction in the cross-modal retrieval. Since the multi-modal data are often located in different embedding spaces, it is reasonable to find a common Hamming space shared by multi-modal data, ensuring efficiency and effectiveness. Inspired by previous observations, various supervised cross-modal retrieval methods [53,54], and unsupervised ones [55,56,57] perform the feature transformation to the Hamming space and capture the semantic relevance. CPAH [54] learns the consistent modality-specific representations and adopts the adversarial learning to enforce inter-modality semantic consistency. Liu et al. proposed a joint-modal distribution similarity Hashing (JDSH) [55] based on DJSRH [56] construct a joint-modal similarity matrix to preserve the cross-modal semantic correlations among instances fully. However, comparing with existing cross-modal hashing retrieval methods, e.g., the DCMH [58] and PRDH [59], our method considers quantization loss and bit balance loss. It further captures semantic relevance and modality invariance by end-to-end learning the joint binary hash code representations between IR video and EEG modalities.

## 3. Materials and Methods

### 3.1. Research Materials

**Figure 1:**
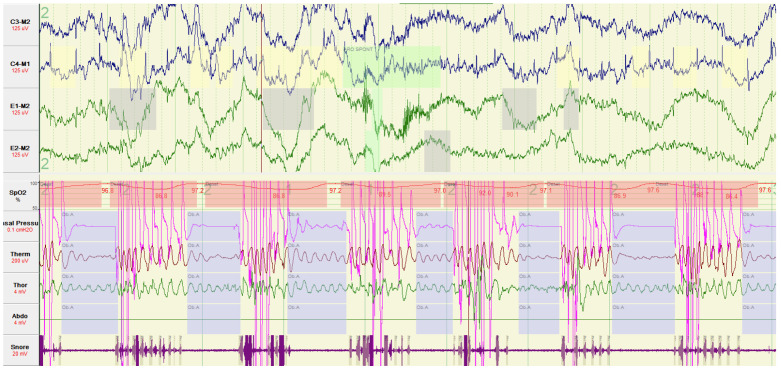
A demonstration of the EEG signals collected by the PSG device.

In our study, we used the $S^{3}VE$ dataset [60], which consists of two parts, the PSG (polysomnography) multiplexed physiological signals and the synchronised infrared video. PSG signals are collected according to the guidelines of the American Academy of Sleep Medicine (AASM). Multiplex signals specifically refer to: EEG (electroencephalogram) (C3M2, C4M1), symmetrical bilateral ECG (electro-oculogram) (E1-M1 and E2-M2), chin muscle EMG (electromyogram), oral and nasal thermistor, nasal pressure, chest and abdominal movements, ECG (electrocardiogram), snoring, body position, bilateral anterior tibial muscle EMG (electromyogram), pulse oximetry and heart rate, and oxygen saturation. In Figure 1, we give several physiological electrical signals as a demonstration, and since we are studying the internal connection between single-lead EEG and IR video, the EEG signals in the following studies are taken from the C3-M2 channel.

**Figure 2:**
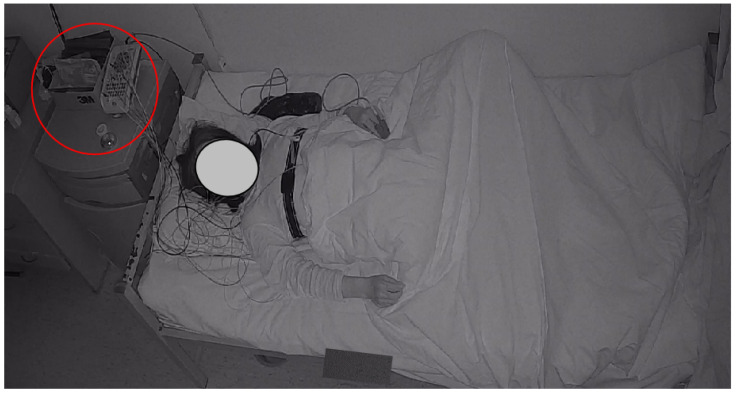
Experimental setting and PSG device. The PSG device is in the red circle on the upper left.

Figure 2 shows a frame from an infrared sleep video with the PSG device in a red circle in the upper left corner. Infrared sleep videos measure 1920 × 1080 and have a frame rate of 25 fps. IR videos capture the patient’s body movements, facial expressions, and respiratory rhythms as they sleep, which are closely related to sleep stage classification and are discussed in articles [6,7,60]. In addition, EEG is the “gold-standard” for sleep stage classification, which provides a theoretical basis for our study.

### 3.2. Overall Framework

**Figure 3:**
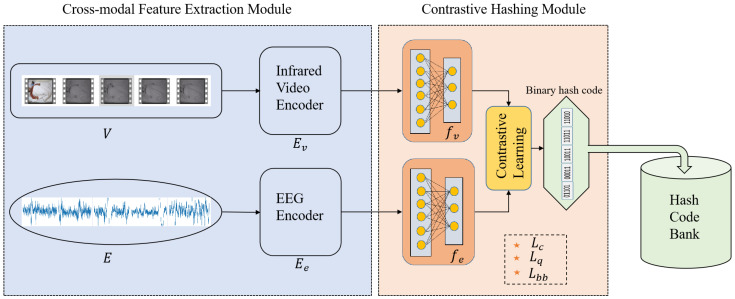
Overview of the proposed Cross-modal Contrastive Hashing Retrieval method. The pipeline of our method consists of two components: Cross-modal Feature Extraction Module that extract deep semantic features from the raw clips of IR videos and EEG modalities; Contrastive Hashing Module that achieve consistency in cross-modal representation and generate unique binary hash code to facilitate storage.

The essential core of our method is to learn discriminative and compact binary codes for IR videos and EEG, such that: (1) representations of the synchronized IR video and EEG signal clip should maintain close semantic consistency—that is, the uni-modal clips should share the same and unique binary code; (2) both the inter- and intra-modality semantic feature spaces should preserve the well-defined metric embedding properties, i.e., the embedded features of semantically similar data pairs should have smaller Hamming distance than others. (3) Each bit of the binary hash code is obtained with equal probability without any bias. To meet the above requirements, as demonstrated in Figure 3. Our method CCHR is composed of the following two modules:

- Cross-modal feature extraction module that provides deep semantic representation for IR video and EEG signal via deep neural networks.
- Contrastive hashing module that generates the instance-level binary hash code of the deep semantic features through cross-modal contrastive learning.

We mainly introduce the above two modules in Section 3.3 and Section 3.4, respectively, and introduce the details of network optimization in Section 3.5.

### 3.3. Cross-Modal Feature Encoders

The purpose of the feature encoder is to firstly compute the semantic features of the raw IR videos and EEG signals and secondly project them to the hashing module. The encoder architecture is shown in Figure 3, where we are given the *i*th synchronized EEG signal $e_{i}$, and the synchronized IR video $v_{i}$ collected from real-world subjects in the hospital. The time duration of $e_{i}$ and $v_{i}$ are both 30 s, that is named clip for instance-level retrieval. The raw data of the two modalities are denoted by *E* and *V*, respectively. Then the overall multi-modal set can be expressed as *S*, and *S* = ${\left\{E,V\right\}}^{N}$, where *N* is the number of pairs of multi-modal datasets. In Figure 3, the IR and EEG feature encoders are represented as $E_{v}$, and $E_{e}$, respectively. It is worth noting that the weights of the IR video and EEG encoders are obtained from the pre-trained network and frozen during the training of the contrastive hashing module. The IR video $v_{i}$ and the synchronized EEG signal $e_{i}$ have a common ground-truth sleep stage annotation $y_{i}$. The annotation is obtained by the clinicians, via analyzing the PSG signals and is regarded as the “gold-standard” annotation of the sleep stage classification. The annotation set consists of five categories: the *W* stage, N1 stage, N2 stage, N3 stage, and *R* stage. Therefore, the IR video encoder $E_{v}$ and EEG encoder $E_{e}$ are pre-trained based on the sleep label set *Y* that learn the sleep stage representations. However, because our work focuses on the instance-level cross-modal retrieval, excessive representation of classes and categories is prone to be detrimental to the retrieval performances. This inspires us to fine-tune the pre-trained networks via the instance-level cross-modal contrastive learning in the contrastive hashing module Section 3.4.

### 3.4. Contrastive Hashing Module

The contrastive hashing module aims at learning hashing functions $f_{v}\left(\cdot \right)$ and $f_{e}\left(\cdot \right)$ to generate binary hash codes from the IR and EEG feature embeddings. To this end, we design three loss objectives, including the cross-modal constrastive loss $L_{c}$, the hashing quantization loss $L_{q}$ and the hashing bit balance loss $L_{bb}$. The overall loss function *L* is a weighted sum of the above three objectives:

(1) $$\min_{B,\theta_{v},\theta_{e}}L=L_{c}+\lambda_{1}L_{q}+\lambda_{2}L_{bb}$$

where *B* is the final hash code corresponding to the two modalities’ clips; $\theta_{v}$ denotes the parameters of the hashing functions $f_{v}\left(\cdot \right)$ and $\theta_{e}$ denotes the parameters of the hashing functions $f_{e}\left(\cdot \right)$. $\lambda_{1}$ and $\lambda_{2}$ are hyper-parameters for the quantization loss and the bit balance loss. The contrastive hashing module is trained by optimizing the Equation (1). and the generated binary hash codes are stored in the hash code bank for cross-modal retrieval. In the inference stage, to retrieve the most relevant EEG clip given a query IR video clip, we compute the Hamming distance between $f_{v}\left(v_{i}\right)$ and the hash codes in the “all-encompassing” hash code bank. The obtained Hamming distances are arranged in the ascending order, and the top-*K* similar EEG clips are the retrieved results. Similarly, when the query EEG clip is given, the Hamming distance between $f_{e}\left(e_{i}\right)$ and the hash code in the hash code bank is calculated. The ranking top-*K* infrared videos are regarded as the retrieval result. Next, we describe the three objectives in details separately.

#### 3.4.1. Cross-Modal Contrastive Loss

Since $f_{v}\left(v_{i}\right)$ and $f_{e}\left(e_{i}\right)$ contain information in different modalities (IR video and EEG), directly enforcing their similarity in feature space does not work well. Instead, we propose a cross-modal contrastive loss to solve this problem. The triplet loss is widely used to learn feature embedding based on the relative similarity of the sampled pairs, such as [61,62]. The goal of the original triplet loss is to assign close distance to pairs of similar samples (positive pair) and long distance to pairs of dissimilar samples (negative pair). Its formula can be expressed as:

(2) $$L_{triplet}={\left[d{\left(x_{a},x_{p}\right)}^{2}-d{\left(x_{a},x_{n}\right)}^{2}+margin\right]}_{+}$$

where ${\left[\;\cdot \;\right]}_{+}=max\left(0,\;\cdot \;\right)$, $d(x_{i},x_{j})$ represents the distance between the samples, such as Euclidean distance, and the margin is a standard relaxation coefficient. Based on Equation (2), assuming that the number of mini-batches during training is *K*, then we can express our cross-modal constrastive loss as:

(3) $$\begin{array}{c} L_{c}=\sum_{k=1}^{K}\{[\alpha -S\left(f_{v}\left(v_{k}\right),f_{e}\left(e_{k}\right)\right)+S{\left(f_{v}\left(v_{k}\right),f_{e}\left(e_{m}\right)\right]}_{+}\\ +[\alpha -S\left(f_{v}\left(v_{k}\right),f_{e}\left(e_{k}\right)\right)+S{\left(f_{v}\left(v_{n}\right),f_{e}\left(e_{k}\right)\right]}_{+}\} \end{array}$$

where α is the margin with a default value of 0.25 and can be tuned on the validation set. *m* and *n* are the index for the hard negatives where $m=argsmax_{m\neq k}S\left(f_{v}\left(v_{k}\right),f_{e}\left(e_{m}\right)\right)$ and $n=argsmax_{n\neq k}S\left(f_{v}\left(v_{n}\right),f_{e}\left(e_{k}\right)\right)$. The purpose of taking *m* and *n* here is to use the information inside the single modality. When an anchor index is selected, it is represented by *k* in the Equation (3). The comparison between the two modalities will be performed at the same time. In detailed, select the index of the EEG modality output that is most similar to the infrared video modality output of the *k*th index, and denote it as *m*; Select the index of the infrared video modality output that is most similar to the EEG modality output of the *k*th index, and denote it as *n*. S(·) is the similarity function in the feature space. we use:

(4) $$\begin{array}{c} S(a,b)=exp(cos(a,b)/\tau )\\ cos\left(a,b\right)=a^{T}b/∥a∥∥b∥ \end{array}$$

It is worth noting that the S(·) selection here cannot be a simple bit-wise subtraction and then observe whether each bit is 0 or 1, because the feature output before quantization is used to calculate the cross-modal contrastive loss here, not after quantization.

#### 3.4.2. Quantization Loss

As long as it is a deep hashing method, such as [63,64], the quantization loss is an unavoidable problem. Quantization loss aims to reduce the difference between continuous binary-like codes and discrete hash values. Our quantization loss is expressed as:

(5) $$L_{q}={∥B-f_{v}\left(v_{i}\right)∥}_{F}^{2}+{∥B-f_{e}\left(e_{i}\right)∥}_{F}^{2}$$

where $f_{v}\left(v_{i}\right)$ and $f_{e}\left(e_{i}\right)$ are binary-like codes for IR video and EEG, respectively, and ${∥\cdot ∥}_{F}$ denotes the Frobenius norm.

#### 3.4.3. Bit Balance Loss

The bit balance loss was first proposed by [65] and it enforces each output neuron to fire with an equal chance. The use of bit balance loss results in obtaining a binary representation that all the bits of the hash code are used equally. We denote the bit balance loss as:

(6) $$L_{bb}={∥f_{v}\left(v_{i}\right)\cdot 1∥}_{F}^{2}+{∥f_{e}\left(e_{i}\right)\cdot 1∥}_{F}^{2}$$

The final binary code update rule is defined as:

(7) $$B=sign(\frac{1}{2}\left(f_{v}\left(v_{i}\right)+f_{e}\left(e_{i}\right)\right)$$

Finally, the output hash codes will be stored in the hash code bank for subsequent retrieval.

The whole optimization process for the cross-modal hashing retrieval network is summarized in Algorithm 1.
**Algorithm 1** Optimization Algorithm**Input** Training set X, hyperparameter $\lambda_{1}$, $\lambda_{2}$
**Output** The weights of the IR video hashing network $\theta_{v}$ and EEG hashing network $\theta_{e}$;      The weights of the IR video encoder $\theta_{E_{v}}$ and EEG encoder $\theta_{E_{e}}$ (If the encoder for both      modes is not frozen);  1: **repeat**  2:             Randomly sample a batch of training data with pairwise synchronised IR sleep         videos and EEG signal;  3:             Compute the outputs of the IR sleep videos encoder $E_{v}$ and EEG encoder $E_{e}$  4:             Compute the outputs of two hashing networks $f_{v}$ and $f_{e}$  5:             Calculate the contrastive hashing loss according to Equation (3)  6:             Calculate the quantization loss $L_{q}$ and the bit balance loss $L_{bb}$ according to         Equations (5) and (7), respectively;  7:             Train the target model by optimizing $L_{c}+\lambda_{1}L_{q}+\lambda_{2}L_{bb}$  8: **until** a fixed number of iterations


### 3.5. Network Details

Here, we describe the details of the networks for each part of the CCHR. As shown in Table 1, we chose a similar architectural network structure to 3D resnet18 as the feature extractor for the IR video and pre-trained it on $S^{3}VE$. We use AttnSleep [5] as the feature extractor for EEG, which is only trained on the EEG C3 lead in the dataset $S^{3}VE$ [60]. The output of the infrared video feature extractor is a 512-dimensional tensor. Still, the output of the EEG feature extractor is a 3000-dimensional tensor, which we reduce to 512 dimensions to be consistent with the infrared video modality. As shown in Table 2, the network structure of the contrastive hashing module of infrared video and EEG are all composed of three fully connected layers with input dimensions of 512, 4096 and K (number of bits), respectively. We use two ReLU and one tanh as their activation functions; and there is a BN layer between the second and third fully connected layers. Note: $fc_{i}$ is the $i^{th}$ fully-connected layer, while BN represents the batch normalization layer. It is important to mention that the CCHR does not rely on a certain type of IR and EEG encoders architecture, and the modality-specific encoders can be replaced with encoders of different architecture.

## 4. Experiments

In this section, we systematically analyze the proposed CCHR and compare it with the latest cross-modal retrieval methods in the $S^{3}VE$ dataset [60]. The remainder of this section is organized as follows. We first describe the dataset we use, $S^{3}VE$, and the partitioning of the dataset. Then, experimental details of our method are reported. Subsequently, a full comparison with state-of-the-art methods is given. Finally, an in-depth experimental analysis and visualisation of our method is presented.

### 4.1. Dataset

Our goal is to use cross-modal hashing retrieval (infrared video modality and EEG modality) to assist sleep doctors in diagnosis and treatment. In contrast, the existing sleep datasets, such as Sleep Heart Health Study (SHSS) [66], Montreal Archive of Sleep Studies (MASS) [67], and SleepEDF-78 [68] are primarily performed with a single EEG modality. There are no other datasets with infrared sleep video data other than the dataset $S^{3}VE$ we collected in [60]. Furthermore, studies have rarely considered the relationship between EEG and infrared video modalities. We collected the synchronized EEG and the IR video signals by Polysomnography (PSG) device from Peking University Third Hospital. The dataset comprises 105 individuals, 82 males and 23 females. They were all suspected of having sleep disorders, such as Obstructive Sleep Apnea (OSA), so the sleep physicians recommended they do sleep monitoring. Only 102 of these samples were selected for the study because the remaining three samples (males) were sleep-deprived and overactive for their own reasons. In these samples, the oldest is 70 years old, the youngest is 7 years old, and the average age is 40. Since Apnea–Hypopnea Index (AHI) is essential in determining the severity of OSA, we also count the AHI of all individuals. As shown in the chart below, there are 30 normal (AHI < 5), 20 mild (5 ≤ AHI < 15), 30 moderate (15 ≤ AHI < 30), and 25 severe (AHI > 30). We selected the lead C3-M2 channel as our EEG modality input. $S^{3}VE$ is a large-scale dataset for sleep stage classification. Similar to the previous work [69], a subset of 134,070 clips from 5 categories are considered. We randomly select 10,000 clips from the subset as the query set and use the remaining clips as the database. There were a total of 20,000 clips from the database are randomly selected for training. Training is performed on the training set, evaluation is undertaken on the retrieval set, and queries are selected on the query set. To demonstrate the effectiveness of our method, we compare CCHR with several state-of-the-art cross-modal hashing retrieval methods using the dataset $S^{3}VE$.

### 4.2. Experiment Configurations

As shown in Table 2, the contrastive hashing network consists of three fully connected layers, the last of which is modified as a hash layer, and in the experiments the hash codes are 16, 32, and 64 bits long. The size of the dataset videos is 1920 × 1080 × 25 fps, to speed up the training, we truncate the key areas and reduce the image size to 320 × 240. It should be noted that our EEG modality feature extractor was adapted to the video modality compared to the standard Attnsleep: the sampling frequency was changed to 128 HZ, the feature extraction was not sufficient to take a zero-completion operation, the number of layers of the multi-headed attention mechanism was changed from 5 to 4, and finally, a fully connected layer was added to change the dimensionality. The hyperparameters $\lambda_{1}$ and $\lambda_{2}$ are selected using a grid search strategy and we set $\lambda_{1}=0.001$, $\lambda_{2}=0.01$. We implement our model on PyTorch and employ the optimizer Adam for optimization, in which the default parameters are used and the learning rate is set to be 0.001. The batch size is set to 256 and total number of training epochs is 150. All experiments are conducted on a single NVIDIA RTX 3090.

### 4.3. Evaluation Metric and Baselines

In our experiments, the mean average precision (MAP) at top *N* is used to measure the quality of obtained hashing codes. Generally, MAP measures the discriminative learning ability of different cross-modal retrieval methods, where a higher MAP indicates better retrieval performance. Specifically, given a query video $x_{v}$, average precision (AP) is defined as:

(8) $$AP\left(x_{v}\right)=\frac{1}{R_{k}}\sum_{k}P\left(k\right)S_{1/0}\left(k\right)$$

$R_{k}$ denotes the number of all relevant videos. P(k) means the cut-off point *k* in the list of retrieved videos. $S_{1/0}\left(k\right)$ is an indicator function that equals 1 if the image returned by the $k_{t}h$ is similar to $x_{v}$, and 0 if the video returned by the $k_{t}h$ is different from $x_{v}$. MAP is the mean AP of all L={1,…,l} queries:

(9) $$MAP=\frac{1}{L}\sum_{l}AP\left(x_{l}\right)$$

Referring to the cross-modal retrieval dataset of the same size, MSCOCO [70], we adopt MAP@1000 for the dataset $S^{3}VE$. In this work, we consider the following deep hashing methods for comparison: DCMH [71], PRDH [59], CPAH [54], DJSRH [56], JDSH [55], and DUCH [51]. Several of the above cross-modal hashing retrieval algorithms, supervised and unsupervised, are considered the most representative and state-of-the-art. We replicated and fine-tuned the $S^{3}VE$ dataset according to the code framework they provided (if the hashing retrieval is supervised, the labels generated for our method are used as references), and the dataset-related settings were kept consistent to ensure the fairness of the finishes.

## 5. Results and Analysis

### 5.1. Results

Table 3 shows the performance of our proposed model CCHR on the dataset $S^{3}VE$ [60] with binary hash code lengths ranging from 16 to 64. For comparison, the performance of the baseline model is also included. As can be seen from the table, the proposed CCHR model outperforms the current SOTA methods by a large margin on the dataset $S^{3}VE$. Compared with the strong opponent DUCH [51], our method CCHR improves the hashing code length of B = 16, B = 32, and B = 64 by 3.5%, 4.5%, and 3.1%, respectively, when IR video is used as the query, and EEG is used as the retrieval gallery. Additionally, our method CCHR improves the hashing code length of B = 16, B = 32, and B = 64 by 3.3%, 3.0%, and 2.9%, respectively, when EEG is used as the query, and IR video is used as the retrieval gallery. We attribute some of this performance improvement to the adaptation of contrast loss to the task context. In most cases, the improvement in algorithm performance is more pronounced when the hash code is shorter and less pronounced when the hash code is long enough. The reason for this is that as the hash code becomes longer, the amount of information contained in the word code becomes larger, and the increase in information brought about by the improvement in the algorithm is relatively diluted. As can be easily seen from Table 3, the retrieval performance of IR video as a query is slightly higher than that of EEG as a query for the same bit length, as will be specified in Section 6.

### 5.2. Ablation Study

To analyze the impact of the different objective losses in our design, we designed several sets of comparison experiments under two retrieval tasks (IR→EEG, EEG→IR). The results of the ablation study are shown in Table 4 for CCHR without quantization loss and bit-balanced loss at B = 16, 32, and 64, respectively. In the table, we can observe that the quantization loss $L_{q}$ in Equation (1) has a more significant impact than the bit-balance loss $L_{bb}$, which is also consistent with our intuition since many hashing retrieval work [72] without bit-balance loss. Overall, the impact of quantization loss on retrieval is approximately 3.6–6%, and the effect of bit-balance loss on retrieval results is about 1.3–2.6%, both of which are icing on the cake. This further illustrates that cross-modal contrastive learning makes infrared video and EEG already learn to represent well.

### 5.3. Analysis

**Figure 4:**
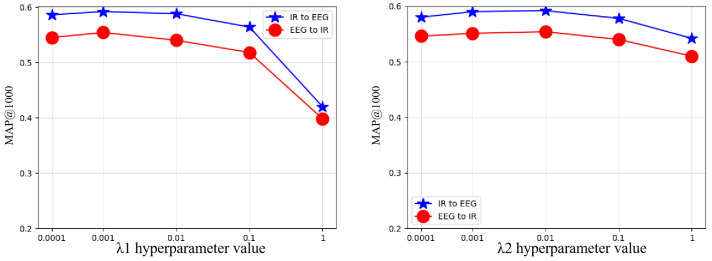
Sensitivity analysis of the proposed CCHR. Cross-modal (IR video and EEG) retrieval performance in terms of MAP@1000 for 64 bits hash codes when varying hyperparameters: $\lambda_{1}$ and $\lambda_{2}$.

In this section, we analyze the impact of different loss function weights on the retrieval performance of the proposed CCHR method. Figure 4 shows the results when selecting different hyper-parameters $\lambda_{1}$ and $\lambda_{2}$ on the $S^{3}VE$ dataset. The results are achieved on MAP@1000 with B = 64 by changing only one hyperparameter and keeping other experimental settings unchanged. We always keep the maximum weight of the contrastive loss as the essential part of the loss function because the purpose of the contrastive loss is to express the semantic consistency of two different modalities. Additionally, the other two-loss functions are to form a better binary hash code. It can be observed that when the coefficients of the three-loss functions are all taken as 1, the performance drops significantly. When hyperparameter $\lambda_{2}$ is fixed and only hyperparameter $\lambda_{1}$ changes, the model performance rises and then falls over a range of values from 0.0001 to 1. The relative performance is maximised when parameter 1 is taken as 0.001, with IR video as query and EEG as query being 0.526 and 0.506, respectively. Additionally, when hyperparameter $\lambda_{1}$ is fixed and only hyperparameter $\lambda_{2}$ changes, the model performance rises and then falls over a range of values from 0.0001 to 1. The relative performance is maximised when parameter 1 is taken as 0.01, with IR video as query and EEG as query being 0.526 and 0.506, respectively.

**Figure 5:**
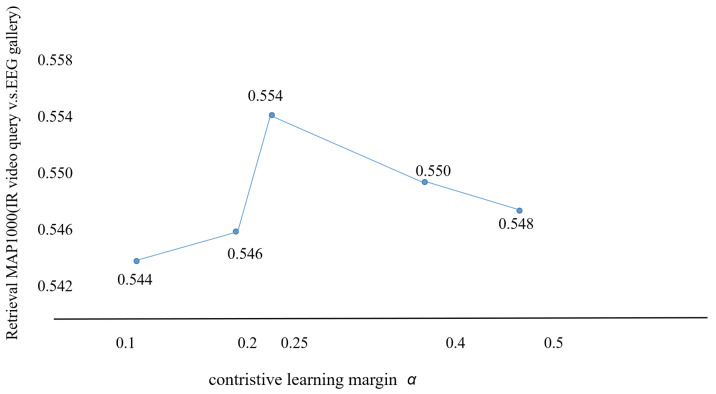
Performances on different values of the contrastive learning margin α (defined in Equation (5)).

We also analyze the sensitivity of the hyperparameter α in the Equation (3) while keeping the other settings of the experiment constant. The results are presented in Figure 5, and we can observe that the results for cross-modal retrieval (IR→EEG, B = 64) rise and then fall with the contrastive learning margin α value increasing, giving a local maximum of 0.554 at α = 0.25. Due to time constraints, we only selected five α values, and we will analyze in more detail using a grid search to find the most suitable margin for the validation set, which is our future work.

For a clear analysis of CCHR, we have listed all the top 100 results of a query. The query was a video of deep sleep with a sleep staging of the N3 stage, which is the sleep classification of most interest to clinical sleep physicians. The top 100 was chosen because some patients have limited stages of deep sleep and too many selections to be a good indicator of individual relevance of the distribution of results. As shown in the Table 5: 5 different patients (A, B, C, D, E) appear in it. Overall, 65% of the search results were for N3 sleep stages, 26% for N2 sleep stages, and 7% for the other three sleep stages. It proves that our search results also show good performance in terms of classification. Of the 35 results for non-N3 sleep classification, more than two-thirds belong to the patient itself, indicating that individuals in the same place are still more likely to be retrieved in the case of inconsistent sleep classification. Of the 66 N3 results retrieved, the patient itself (A) accounted for the vast majority, All of which were ranked very highly, showing that the same sleep stage in the same sample was the most likely to be retrieved.

## 6. Discussion

**Figure 6:**
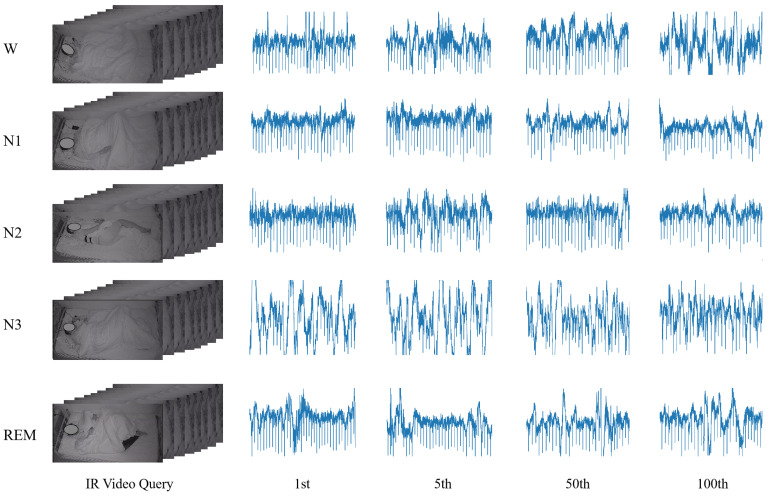
IR video → EEG retrieval results obtained when hash code length is 64 for $S^{3}VE$ dataset [60]. Regarding the EEG, we show the raw signal output by the PSG device, with some artifacts and noise, which we will analyze in detail in Section 6.

**Figure 7:**
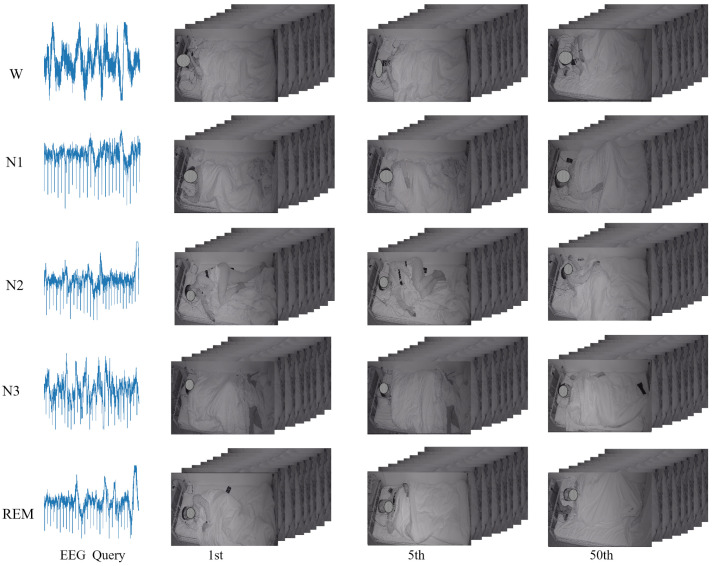
EEG → IR video results obtained when hash code length is 64 for $S^{3}VE$ dataset [60]. Regarding the EEG, we show the raw signal output by the PSG device, with some artifacts and noise, which we will analyze in detail in Section 6.

To visualize the retrieval results of CCHR under each sleep stage, we show the IR sleep video as the query and the EEG signal as the query in Figure 6 and Figure 7, respectively. In Figure 6, we give the EEG signals for the 1st, 5th, 50th, and 100th under each of the five sleep classifications. What can be seen is that the retrieved EEG signals are highly similar. They all match the characteristics of the respective modalities, e.g., the retrieved EEG results for W are high in frequency and have a β wave shape. In contrast, the retrieved EEG results for N3 are low in frequency since N3 is in deep sleep and is relatively stable. This is reflected in the video as there is no massive body movement and the respiratory rhythm is very calm. In addition, the R retrievals all showed sawtooth waves. The video on the R stage is often accompanied by small body movements, and rapid eye movements.

In Figure 7, an example analysis of the retrieval of the corresponding infrared sleep video is shown using the five EEG waveforms as query. For the 1st, 5th, and 50th results in the W stage video, the patient in the video is looking towards the mobile phone and is accompanied by more substantial body movements during these 30 s. In N2 and N3, it can be observed that the specific body position of the 1st and 5th outcomes are different, and this is often the case in other sleep stages, suggesting that the correspondence between the two modalities manifests itself in changes in the trend rather than in specific movements and gestures. The reflection in the video is that deep semantic information is learned rather than at the pixel level.

Some artifacts and noise can be observed in the EEG signals in Figure 6 and Figure 7, where we visualize the raw EEG signals received by the PSG device. Regarding artifacts and noise, firstly, we install the electrodes under the standard PSG method of Compumedics USA in a suitable position, degrease the skin, apply the conductive paste, and minimize the artifacts and noise. Artifacts and noise have unavoidable systemic reasons, such as body movement and electrode factors, for artifacts and noise to be noticed and to minimize their existence. In clinical diagnosis, the physician’s experience is significant, and the influence of artifacts and noise can be relatively reduced by post-processing the PSG software, such as filtering. In addition, the infrared video contains body movement information that can help us distinguish artifacts. Secondly, we also use extraction of the common components of the EEG channels to eliminate them as artifacts, and a similar approach is shown to work in [73]. One module of the EEG feature extractor is the adaptive feature recalibration (AFR) modeling of correlations between features to enhance feature learning. Another module in our EEG feature extractor is the temporal context encoder (TCE) that deploys a multi-head attention with causal convolutions to efficiently capture the temporal dependencies in the extracted features. The temporal correlation of noise and artifacts is much worse than that of EEG signals, and this step filters out some artifacts and noise. In the system approach, we also use a contrastive learning method, which is more noise tolerant than normal deep learning algorithms due to the contrast between features. Finally, to illustrate the accuracy of our feature extractor and our data can be processed for the evaluation of sleep conditions, we train the feature extractor using sleep classification labels (*W*/N1/N2/N3/REM). We achieve an accuracy of 81.3% (C4-M1) on our dataset, which is sufficient for sleep quality evaluation.

It is worth discussing that, in the results in Table 3, the accuracy of retrieving EEG signals using IR video as a query is slightly higher than retrieving IR videos using EEG signals as a query. We review many infrared sleep videos and EEG signals and analyze the reasons for this. We believe that, probably due to the infrared video being more specific, it contains more information, such as expressions, large body movements, small body movements, breathing rhythms, rapid eye movements, and other information. When specific movements occur in IR videos, they are often accompanied by a switch in the sleep stage or a change in the EEG waveform, so they have good correspondence. However, the EEG signal as a query is more sensitive than the IR sleep video but less diverse than the video. The EEG often has small changes that are difficult to reflect in the IR sleep video, which can easily lead to misinterpretation. In summary, this means that the two modalities have “different thresholds of ease of perception”, which is caused by a difference between the modalities that cannot be eliminated but only minimized.

In the future, we want to optimize the retrieval algorithm further and make it into a telemedicine assistance system, focusing on solving the problem of too many similarities in deep sleep videos, resulting in too similar retrieval results due to the confidentiality of medical data. We plan to introduce federal learning to enable different hospitals in different regions to participate in cross-modal hash retrieval. Finally, the collection of sleep data is very labor-intensive, and we would like to improve the domain adaptation capability of the existing algorithms.

## 7. Conclusions

In this paper, we propose a novel cross-modal (IR video and EEG) contrastive hashing retrieval method, namely CCHR. Our approach aims to use the internal link between EEG signals and infrared video to build a new idea for telemedicine, allowing patients to receive a relatively accurate sleep classification at home. We attribute the promising performance to two aspects: first, consistent representations between modalities are formed through contrastive learning of hard negative samples. Second, we use quantization loss and bit balance loss to obtain better binary hash codes. Two modules of our model are plug-and-play and replaceable. Extensive experiments have shown that CCHR significantly outperformed existing cross-modal hashing retrieval methods on dataset $S^{3}VE$. Finally, we would like to point out that cross-modal retrieval of IR video and EEG has essential meaning for human sleep research, which is also our future research direction.

## 8. Patents

This section is not mandatory, but may be added if there are patents resulting from the work reported in this manuscript.

## Figures and Tables

**Table 1:** Network architectures of the IR video feature extractor $E_{v}$; each convolutional layer is followed by batch normalization and a ReLU. (F is the number of the channels, and N is the number of blocks in each layer).

Model	Block	*conv1*	$\mathrm{conv}2_{x}$		$\mathrm{conv}3_{x}$		$\mathrm{conv}4_{x}$		$\mathrm{conv}5_{x}$	
			F	N	F	N	F	N	F	N
$E_{v}$	basic	conv, 7 ∗ 7 ∗ 7, 64 temporal stride 1 spatial stride 2	64	2	128	2	256	2	512	2

**Table 2:** Specific structure of the infrared sleep modality and EEG modality hashing module.

Module	Layer	Activation	Output Size
IR video contrastive hashing network	fc1	RELU	512
	fc2	ReLU	4096
	BN	/	/
	fc3	tanh	K
EEG video contrastive hashing network	fc1	ReLU	512
	fc2	ReLU	4096
	BN	/	/
	fc3	tanh	K

**Table 3:** MAP@1000 comparison with different state-of-the-art cross-modal hashing retrieval methods on the dataset $S^{3}VE$.

Task	Method	B = 16	B = 32	B = 64
	DCMH [71]	0.446	0.467	0.510
	PRDH [59]	0.462	0.490	0.538
IR video query	CPAH [54]	0.497	0.511	0.559
vs.	DJSRH [56]	0.485	0.510	0.557
EEG gallery	JDSH [55]	0.478	0.502	0.550
	DUCH [51]	0.508	0.522	0.574
	CCHR (proposed)	**0.526**	**0.546**	**0.592**
	DCMH [71]	0.401	0.421	0.451
	PRDH [59]	0.386	0.426	0.458
EEG query	CPAH [54]	0.447	0.460	0.492
vs.	DJSRH [56]	0.485	0.491	0.521
IR video gallery	JDSH [55]	0.481	0.490	0.531
	DUCH [51]	0.490	0.499	0.538
	CCHR (proposed)	**0.506**	**0.514**	**0.554**

**Table 4:** Ablation studies on the hashing loss of our cross-modal contrastive hashing retrieval. “CCHR w/o $L_{q}$” indicates our CCHR without the quantization loss.

Task	Method	B = 16	B = 32	B = 64
IR video query	CCHR w/o $L_{q}$	0.488	0.494	0.533
vs.	CCHR w/o $L_{bb}$	0.512	0.520	0.575
EEG gallery	CCHR	0.526	0.546	0.592
EEG query	CCHR w/o $L_{q}$	0.470	0.485	0.517
vs.	CCHR w/o $L_{bb}$	0.493	0.502	0.537
IR video gallery	CCHR	0.506	0.514	0.554

**Table 5:** Analysis of the retrieval results of an infrared sleep video (sleep stage of N3, hash code length of 64). We bold the results belonging to N3, italicise those belonging to N2 and underline the rest.

Rank	Top 100 Retrieval Results									
1–10	**A-N3**	**A-N3**	**A-N3**	**A-N3**	*A-N2*	**A-N3**	**A-N3**	*A-N2*	**A-N3**	**D-N3**
11–20	**D-N3**	**A-N3**	*A-N2*	**A-N3**	**A-N3**	**B-N3**	**A-N3**	**A-N3**	*A-N2*	*A-N2*
21–30	**A-N3**	**A-N3**	*B-N2*	*B-N2*	**A-N3**	**A-N3**	**B-N3**	**B-N3**	**B-N3**	*C-N2*
31–40	**A-N3**	**A-N3**	**A-N3**	*A-N2*	*A-N2*	*A-N2*	**A-N3**	**A-N3**	**A-N3**	**C-N3**
41–50	**C-N3**	*A-N2*	**A-N3**	**A-N3**	**A-N3**	*C-N2*	**E-N3**	**E-N3**	**E-N3**	**E-N3**
51–60	**E-N3**	**B-N3**	*B-N2*	**B-N3**	**B-N3**	*A-N2*	*A-N2*	**C-N3**	**C-N3**	**C-N3**
61–70	**C-N3**	**C-N3**	**C-N3**	**C-N3**	**B-N3**	**B-N3**	**D-N3**	**D-N3**	**D-N3**	*A-N2*
71–80	*A-N2*	*A-N2*	**B-N3**	**B-N3**	**B-N3**	D-R	A-R	D-N1	A-N1	*B-N2*
81–90	**A-N3**	**A-N3**	**A-N3**	**A-N3**	**A-N3**	**A-N3**	*A-N2*	A-W	**C-N3**	*A-N2*
91–100	*A-N2*	*A-N2*	**E-N3**	**E-N3**	**E-N3**	A-N1	A-N1	*A-N2*	*C-N2*	*C-N2*

## Data Availability

We validated our method by an open-access dataset, namely, the $S^{3}VE$ dataset accessed on 26 July 2022 (https://ieee-dataport.org/documents/sssvedataset). The dataset generated during and/or analyzed during the current study are available from the corresponding author on reasonable request.

## Abbreviations

The following abbreviations are used in this manuscript:

EEG	Electroencephalography
PSG	Polysomnography
OSA	Obstructive Sleep Apnoea
AASM	American Academy of Sleep Medicine
REM	Rapid Eye Movement
SVM	Support Vector Machines
RF	Random Forests
ECG	Electrocardiogram
EMG	Electromyogram
IR	Infrared
AHI	Apnea–Hypopnea Index
MAP	Mean Average Precision
SOTA	State of the art
SHSS	Sleep Heart Health Study
MASS	Montreal Archive of Sleep Studies
